# Kinship Analysis Confirms Tolerant Galapagos Mockingbirds Are a Source of Nest Flies That Threaten Darwin's Finches

**DOI:** 10.1111/mec.70334

**Published:** 2026-04-02

**Authors:** Matthew M. Waller, Sarah E. Bush, Andrew D. Sweet, Dale H. Clayton

**Affiliations:** ^1^ School of Biological Sciences University of Utah Salt Lake City Utah USA; ^2^ Department of Biological Sciences Arkansas State University Jonesboro Arkansas USA

**Keywords:** parasite, *Philornis downsi*, tolerant host, transmission, vulnerable host

## Abstract

Host species have evolved different strategies for dealing with parasites. ‘Vulnerable’ hosts, which suffer reduced fitness from parasites, are under selection to evolve defences. ‘Tolerant’ hosts, by contrast, avoid reductions in fitness by mitigating parasite damage. When tolerant and vulnerable hosts co‐occur, tolerant hosts may pose an indirect threat to vulnerable hosts by serving as a source of parasite infestation. Avian vampire flies (*Philornis downsi*) are introduced parasites of Galapagos mockingbirds ( *Mimus parvulus* ) and Darwin's finches. Mockingbirds are relatively tolerant, while finches are more vulnerable. We tested the indirect threat hypothesis using a population genomics approach to estimate transmission of vampire flies from mockingbirds to finches on Santa Cruz Island, Galapagos. Mockingbirds nested earlier than finches, which increased their potential threat to finches. We sequenced 136 whole genomes of avian vampire flies and did kinship analysis to compare the relatedness of flies in mockingbird and finch nests over the course of the breeding season. Our data show that flies in source mockingbird nests are more often related to flies in finch nests than to flies in mockingbird nests later in the season. This pattern may be explained by the distance between nests, as finch nests are often closer to mockingbird nests than mockingbird nests are to each other. These results are consistent with the hypothesis that tolerant mockingbirds pose an indirect threat to more vulnerable finches.

## 1 Introduction

Hosts vary in how they defend themselves against parasites. Vulnerable hosts suffer reduced fitness and are thus under selection to evolve defence mechanisms that reduce parasite load (Miller et al. 2005). Tolerant hosts do not suffer reduced fitness because they are capable of mitigating parasite damage without the need to reduce parasite load (Roy and Kirchner 2000; Medzhitov et al. 2012). Because tolerant hosts can support larger numbers of parasites, they may represent an indirect threat to more vulnerable hosts (VanderWaal and Ezenwa 2016; Martin et al. 2019). For example, tolerant grey squirrels ( *Sciurus carolinensis* ) in the United Kingdom increased the prevalence of parapoxvirus in the host community and contributed to the decline of red squirrels ( *Sciurus vulgaris* , Tompkins et al. 2003). In extreme cases, tolerant hosts may contribute to the decline of critically endangered species by increasing the prevalence of pathogens. For example, common eastern froglets ( *Crinia signifera* ), which are tolerant hosts of *Batrachochytrium dendrobatidis* fungus, increase the prevalence of this lethal fungus in endangered northern corroboree frogs ( *Pseudophryne pengilleyi* , Scheele et al. 2017).

Although tolerant hosts can be a source of parasites that increase transmission to vulnerable hosts, this is not necessarily the case. Tolerant hosts maintain fitness in the face of infestation and may thus be a higher quality resource for parasites than vulnerable hosts. Consequently, parasites may preferentially infest tolerant hosts. For example: *Philornis torquans* nest flies infest more than 20 species of Argentinian birds (Manzoli et al. 2013). However, they prefer to parasitize tolerant great kiskadees ( *Pitangus sulphuratus* ) rather than more vulnerable hosts, such as greater thornbirds ( *Phacellodomus ruber* ) (Manzoli et al. 2013, 2021). Thus, rather than acting as a source of flies, tolerant hosts can draw parasites away from more vulnerable hosts.

Tolerant hosts may also display lower pathology in response to parasites, further reducing transmission to vulnerable hosts (Henschen and Adelman 2019). For example, house finches ( *Haemorhous mexicanus* ) that are tolerant to *Mycoplasma gallisepticum* bacteria have smaller lesions and deposit fewer bacteria on bird feeders, reducing transmission to new hosts (Adelman et al. 2013). The net effect of tolerant hosts on transmission, either positive or negative, is context dependent and influenced by a variety of factors, such as parasite preference (Manzoli et al. 2021; Tadiri et al. 2021) and host pathology (Henschen and Adelman 2019).

The avian vampire fly (*Philornis downsi*) is an invasive parasitic nest fly of tolerant and vulnerable species of landbirds in the Galapagos Islands (Knutie et al. 2016; McNew et al. 2019). Adult flies are not parasitic but feed on decaying organic matter. Each mature female fly lays an average of 5 eggs (range 1–24 eggs) in a host nest (Dudaniec et al. 2010). Upon hatching, the larval flies feed on the blood and tissues of nestling birds. Following three larval stages, third instar larvae pupate in the nest material. Adult flies emerge from the pupae 9–12 days later (Fessl et al. 2006). Overall, the *P. downsi* life cycle requires a minimum of 29 days to complete (Causton et al. 2019).

Avian vampire flies were first collected in the Galapagos in the 1960s (Causton et al. 2006). Since then, the fly has spread to most of the Galapagos Islands (Wiedenfeld et al. 2007). These vampire flies parasitize many landbirds in the Galapagos, including at least eleven species of Darwin's finches (Fessl et al. 2018). Avian vampire flies have been implicated in the decline of two critically endangered species of Darwin's finches, the mangrove finch ( *Camarhynchus heliobates* ) and the medium tree finch ( *Camarhynchus pauper* ) (Fessl et al. 2010; O'Connor et al. 2009). Most species of Darwin's finches are vulnerable to vampire flies, which cause up to 100% nestling mortality (Koop et al. 2011). In some cases, finches are less vulnerable; for example, urban populations of small ground finches ( *Geospiza fuliginosa* ) with protein‐rich diets show increased expression of immune genes and greater fledging success (Knutie et al. 2024). However, since most Darwin's finches do not live in urban areas, they are vulnerable to the flies (Koop et al. 2011, 2013).

Galapagos mockingbirds ( *Mimus parvulus* ), by contrast, are relatively tolerant hosts of avian vampire flies and may suffer little or no reduction in fledging success (Knutie et al. 2016). Infested mockingbird nestlings increase their rate of begging in response to parasitism, and the parents respond by feeding them more, which appears to mitigate parasite damage. In typical years, fledging success does not differ between nests with and without flies. In dry years, however, when food is more limited, parents cannot increase provisioning, resulting in a loss of tolerance and reduced fledging success of infested mockingbird nests (McNew et al. 2019).

Flies that emerge from mockingbird nests may lay eggs in finch nests; thus, mockingbirds may be an important source of flies that infest Darwin's finches. Mockingbirds often nest earlier in the season than finches and have larger nests, and thus more flies than finches (Knutie et al. 2016; McNew et al. 2019). In dry years when mockingbirds lose tolerance, the number of flies emerging from their nests is similar to the number observed in wet years (McNew et al. 2019). Thus, mockingbird nests may pose an indirect threat to finches in both wet and dry years.

The indirect threat hypothesis is not a foregone conclusion. Flies in the nests of finches in one breeding season might have originated from the nests of finches breeding the previous season. Vampire flies can live for more than 8 months, which exceeds the time between subsequent host breeding seasons (Causton et al. 2019; Bulgarella et al. 2022). Flies may also disperse over long distances. For example, recent work by Basnet et al. (2025) suggests ongoing gene flow between populations of flies on different islands. Fly dispersal over long distances may be human mediated: *P. downsi* has been trapped on boats travelling between islands (Lomas 2008).

Identifying sources of parasites under natural conditions is challenging because, given their small size, individual parasites cannot be marked and followed during transmission from one host species to another. However, genomic‐based kinship approaches have recently been developed to infer dispersal and quantify transmission over fine scales. For example, Jasper et al. (2019) quantified intergenerational dispersal distances by identifying close kin among mosquitoes in a single apartment complex. They used genome‐wide SNPs to identify pairs of closely related mosquitoes and compared the spatial distribution of close relatives (e.g., siblings, first cousins) to infer dispersal. Kinship analysis can also be used to investigate the movement of parasites among hosts. The presence of closely related parasites in two species of hosts is consistent with transmission between hosts. The timing of infestation can also be used to infer the direction of transmission. If one host species is infested earlier in the season than a second host species, then the first host species is likely to be the source of transmission to the second host species.

Here, we used kinship analysis to infer whether avian vampire flies may be moving from mockingbird nests to finch nests. We quantified the relatedness of flies within nests and between nests separated by the minimum generation time of avian vampire flies to infer the frequency and direction of transmission between mockingbird nests and finch nests. Presence of related flies in nests of early nesting mockingbirds and later nesting finches would be consistent with transmission of flies from mockingbird nests to finch nests. We used this approach to explore the hypothesis that mockingbirds are a source of vampire flies that infest Darwin's finches.

## 2 Materials and Methods

### 2.1 Study Site and Sampling

**FIGURE 1:**
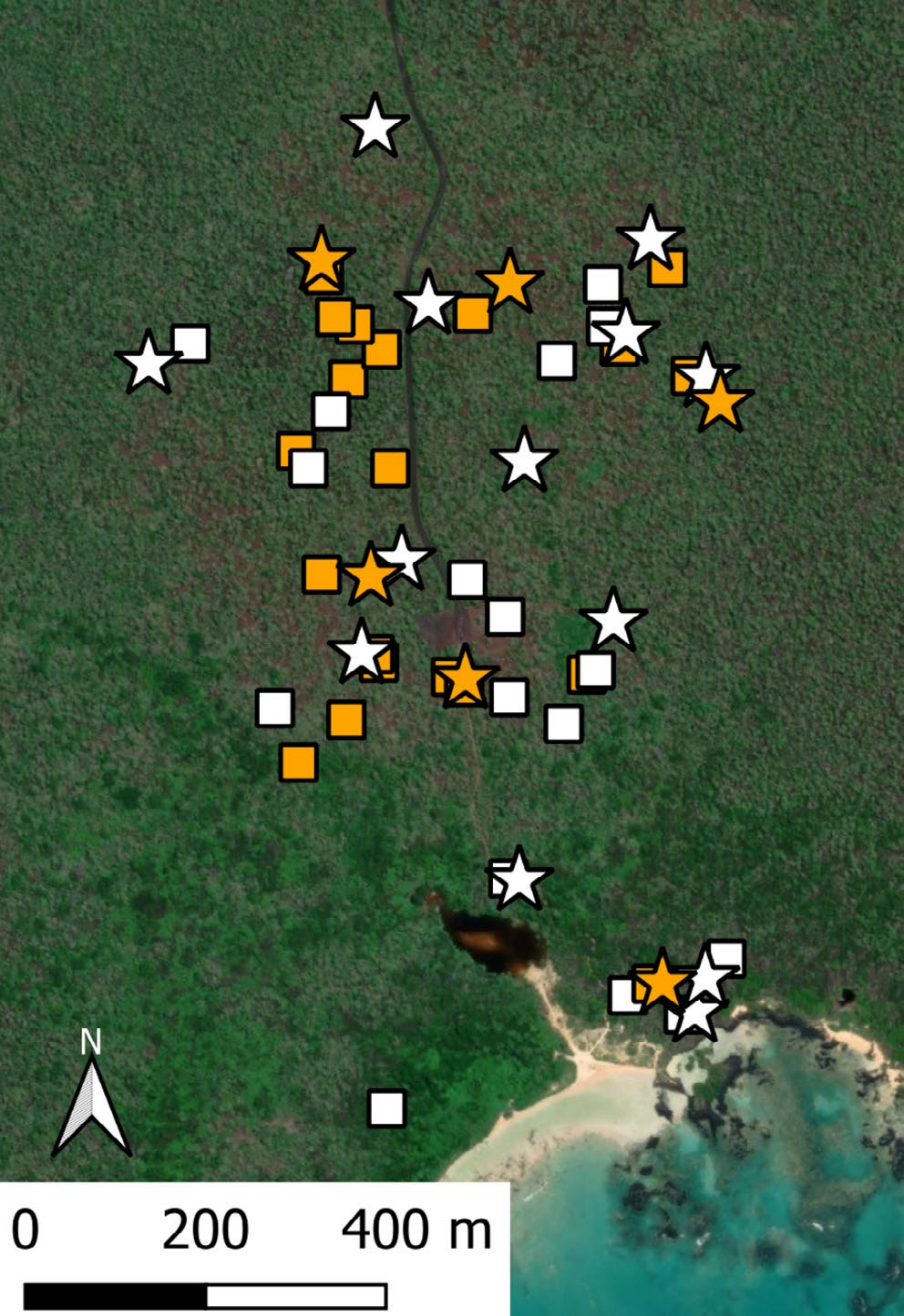
Study site (El Garrapatero, Santa Cruz Island) with nest locations. White symbols represent the 30 mockingbird nests we monitored; orange symbols represent the 26 finch nests we monitored. Stars show nests from which flies were sampled for sequencing; squares show other nests. The map was created in QGIS 3.38.1 software using satellite imagery from ESRI World Imagery (copyright Maxar 2021).

We did field work February 5th–April 28th 2022 at El Garrapatero on Santa Cruz Island in the Galapagos archipelago, Ecuador. El Garrapatero is a 1.5 × 1.5 km area in the arid coastal zone, approximately 10 km east of the town of Puerto Ayora (Figure 1). We searched the site for active nests of Galapagos mockingbirds and Darwin's finches, which are the most abundant hosts of avian vampire flies at this location. Both mockingbirds and finches build conspicuous nests in giant prickly pear cacti (*Optunia galapageia*) or small *Acacia rorudiana* trees. Most nests were located before the eggs hatched, but a small number were discovered soon after eggs hatched. We continued to search the field site for new nests throughout the study.

### 2.2 Nest Monitoring

We checked mockingbird and finch nests every 2–3 days with a borescope (Teslong NTS300) to determine when eggs were laid, when they hatched, and when nestlings died or disappeared from the nest as a result of fledging or predation. Once the nestlings disappeared, we collected and dissected each nest to quantify the number of flies. Collection of nests did not impact subsequent breeding attempts because mockingbirds do not re‐use nests and finches only rarely re‐use them (Grant 1999; Knutie et al. 2016). For each nest we counted the number of second and third larval instars, as well as the number of pupae and eclosed pupal cases. First instar larvae are small and often found in birds' nares (nostrils), making it difficult to accurately quantify the number of first instar larvae. We tested for a difference in the number of flies in mockingbird and finch nests using a Mann–Whitney U test. Statistical analyses were run in R, version 4.4.1. (R Core Team 2024).

**FIGURE 2:**
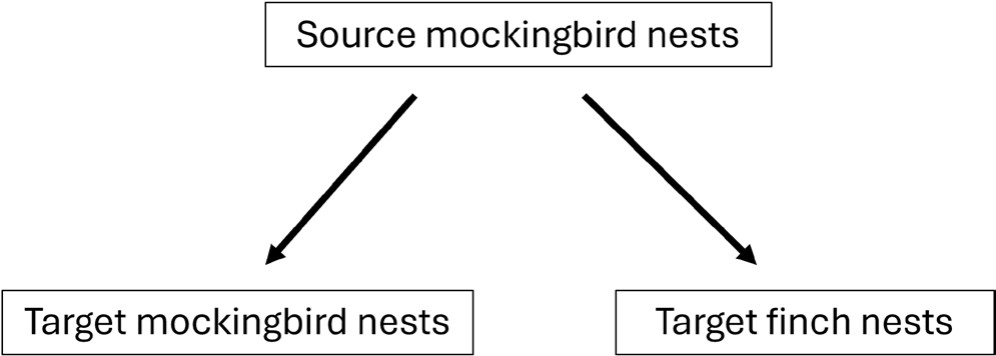
Hypothetical transmission of flies between source nests (early season mockingbirds) and target nests (late season mockingbirds or finches).

**FIGURE 3:**
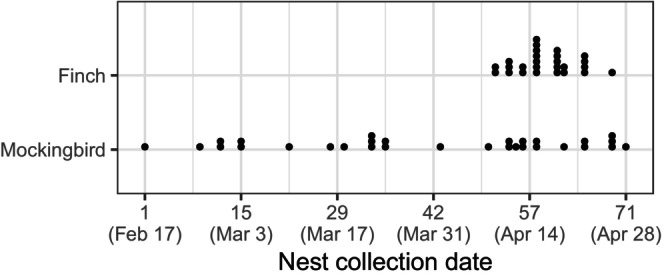
Relative timing of finch and mockingbird nests at El Garrapatero in 2022. Points are the dates nests were collected after the death or disappearance of nestlings. Day 1, February 17th was the first day that a nest was collected.

At least 10 immature flies (larvae and/or pupae) from early season mockingbird nests (Figures 2 and 3) were preserved in 95% EtOH for genomic analysis. We preserved 20% of the third instar larvae and pupae from nests containing more than 50 flies. We preserved all first and second instar larvae, as they cannot complete development after being removed from the nest. Non‐preserved pupae and third instar larvae, together with the dissected nest material, were placed in a container that we returned to the nest tree, which allowed the pupae to eclose and third instar larvae to pupate and potentially eclose. At the end of the field season, we re‐examined the nest material from 4 containers to count the number of eclosed pupae to check that flies could finish development after nest dissection. Of the 376 flies (281 pupae and 95 3rd instar larvae) in the containers, 234 eclosed. All larvae and pupae from mockingbird nests collected during the last 2 weeks of fieldwork were preserved in 95% EtOH, because there was not sufficient time for these flies to finish development and lay eggs in finch nests (Figure 3). All larvae and pupae from finch nests were preserved in 95% EtOH.

### 2.3 DNA Extraction and Genotyping

The mockingbird breeding season was about three times longer than the finch breeding season, providing ample opportunities for mockingbirds that nested early in the season to be a source of flies that infest later nesting mockingbirds and finches (Figures 2 and 3). Our sampling represented nests throughout the entire breeding season; specifically, we sequenced flies from a total of 13 mockingbird nests and six finch nests. Nests chosen for fly sequencing also provided broad spatial representation (Figure 1). We sequenced enough flies from each sampled nest to explore the relatedness of flies both within and among nests (Appendix S1). We attempted to extract DNA from 8 flies per nest (although extraction was not always successful). In total, we extracted DNA from 136 *P. downsi* for a mean of 7.2 flies per nest (minimum = 5 per nest; maximum = 8 per nest). Consequently, we sequenced an average of ~12% of the flies within single nests (range 5%–28%). Previous work by Dudaniec et al. (2010) has shown that the relatedness of flies within nests is not affected by percent of individuals sequenced from each nest. We used Qiagen DNeasy Blood and Tissue kits to extract DNA following a modified protocol (Appendix S2). Libraries were constructed and sequenced on the Illumina X platform by the University of Utah High Throughput Genomics shared resource, obtaining 150 bp paired‐end reads. After sequencing, barcoded reads were filtered and trimmed using the trimmomatic programme version 0.39 (Bolger et al. 2014). We retained reads with a minimum length of 75 and a quality score of at least 15 with a sliding window approach of 4 bases. Filtered paired‐end reads were aligned to the *P. downsi* reference genome assembly (Romine et al. 2022) with Bowtie2 version 2.2.9 using end to end alignment (Langmead and Salzberg 2012). We reconstructed a total of 136 *P. downsi* whole genomes.

We identified SNPs and filtered with VCFtools version 0.1.15 (Danecek et al. 2011) and BCFtools version 1.16 (Li 2011; Danecek et al. 2021). We retained biallelic SNPs with less than 5% missing genotypes and with minor allele frequencies > 0.02. We filtered to remove SNPs out of Hardy–Weinberg equilibrium with a *p*‐value cutoff of 0.05. We filtered for linkage disequilibrium using a sliding window approach with a length of 1000 and a threshold of 0.2. In total, we had 428,732 SNPs for downstream analyses.

### 2.4 Quantifying Relatedness

We used the programme NgsRelate version 2 (Hanghøj et al. 2019) to calculate kinship coefficients, *φ*, (Jacquard 1974) of flies within and between nests. The coefficient of kinship of two individuals, *φ*, is the probability that two randomly selected alleles—one per individual at the same locus—are identical by descent (Jacquard 1974). The theoretical mean values for close kinship categories are: 1st degree *φ* = 0.25, 2nd degree *φ* = 0.125, 3rd degree *φ* = 0.0625. Thus, flies with *φ* > 0.1875 are most likely first‐degree kin (full siblings or parent/offspring). Flies with 0.1875 > *φ* > 0.0938 are most likely second‐degree kin (half‐siblings or avuncular, e.g., nephew/uncle). Flies with 0.0938 > *φ* > 0.0469 are most likely third‐degree kin (1st cousins or half‐avuncular, e.g., offspring of a half‐sibling) (Schmidt et al. 2023).

To infer the direction of transmission between nests, we restricted pairwise calculations of kinship coefficients to flies collected at least 29 days apart, which is the minimum fly generation time (Causton et al. 2019). Nests collected at least 29 days earlier than other nests were potential sources of flies (‘source nests’; Figure 2). Nests collected at least 29 days after other nests were potential targets of flies (‘target nests’; Figure 2). Since finches bred later in the season and were collected within a 29 day period, all finch nests were considered targets (Figure 3). Source nests were early breeding mockingbirds; later breeding mockingbirds were considered targets (Figures 2 and 3).

We calculated a kinship coefficient, *φ*, between flies within source mockingbird nests. Since our genotyping methods required destructive sampling of some flies from each nest, our ability to track fly lineages assumes that siblings of the sampled fly individuals will survive and reproduce in later nests (Appendix S1). Other studies have shown that each female *P. downsi* deposits an average of five eggs per host nest (Dudaniec et al. 2010). Calculating the within‐nest kinship coefficient allows us to determine the relatedness of flies within the source mockingbird nests in this study.

We calculated a kinship coefficient, *φ*, between each fly from a source mockingbird nest and each fly from a target nest (mockingbird or finch). We assigned pairwise relatedness bins for 1st–3rd degree kin based on expected *φ* as described above. All first‐degree kin were full siblings (since flies were collected before they were old enough to breed, there were no parent‐offspring comparisons). Second‐degree kin were half‐siblings, or the offspring of a full sibling. Third‐degree kin were offspring of a half‐sibling or 1st cousins. Flies with *φ* < 0.0469 were considered unrelated.

We calculated the proportion of flies from target finch nests that were related to at least one fly from source mockingbird nests. We also calculated the proportion of flies from target mockingbird nests that were related to at least one fly from source mockingbird nests. We compared the proportion of related flies between source mockingbird nests and target finch nests to the proportion of related flies between source mockingbird nests and target mockingbird nests with a two‐sample test for equality of proportions.

## 3 Results

We collected a total of 30 mockingbird nests and 26 finch nests (Figure 1). The latter included 16 medium ground finch nests ( *Geospiza fortis* ), 2 small ground finch nests ( *Geospiza fuliginosa* ), 5 cactus finch nests ( *Geospiza scandens* ) and 3 vegetarian finch nests ( *Platyspiza crassirostris* ).

**FIGURE 4:**
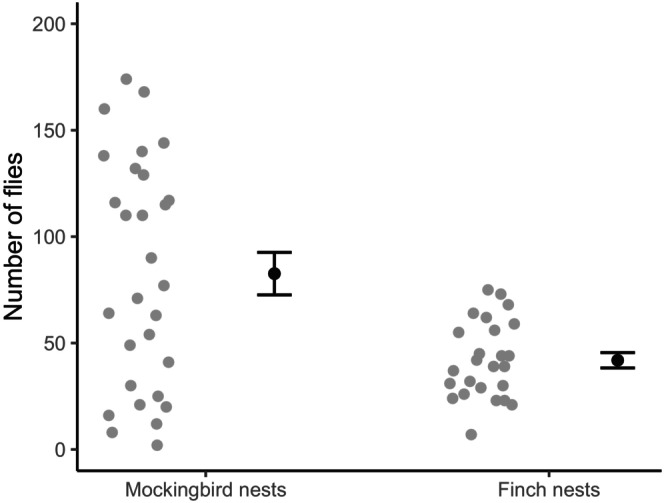
Grey points show the number of flies in each of 29 infested mockingbird nests and 25 infested finch nests. Also shown is the mean (±SE) number of flies for each category of nests. Mockingbird nests had significantly more flies than finch nests (see text).

Mockingbirds started nesting earlier than finches, with the first mockingbird eggs hatching 46 days before the first finch eggs (Figure 3). Mockingbirds continued to nest throughout the study period. Flies were found in all but one mockingbird nest and one finch nest (Table 1). Mockingbird nests were infested with significantly more flies than finch nests (Figure 4, Mann–Whitney test, *p* = 0.013).

**TABLE 1:** Prevalence and mean number of avian vampire flies in mockingbird and finch nests.

	Mockingbird nests	Finch nests
Prevalence	96.7% (29/30 nests)	96.2% (25/26 nests)
Mean (±SE) number of flies per infested nest	82.62 ± 10.00	41.92 ± 3.61

We sampled 41 flies from ‘source’ mockingbird nests. We calculated kinship coefficients for each fly with other flies sampled from the same nest. In all, we calculated 121 kinship coefficients; 19 of these coefficients indicated second‐degree relatives; 15 indicated third‐degree relatives. Of 41 flies sampled from source mockingbird nests, 22 had at least one second‐degree relative (presumably half‐siblings). No flies from source mockingbird nests appeared to be first‐degree relatives (full siblings).

We calculated 1641 kinship coefficients for pairs of flies from ‘target’ and source mockingbird nests. Each fly from a target mockingbird nest was compared to an average of 21.88 flies (range: 7–41) from source mockingbird nests to calculate kinship coefficients. One coefficient indicated first‐degree relatives (presumably full siblings); four coefficients indicated second‐degree relatives (presumably avuncular); 50 coefficients indicated third‐degree relatives (presumably half‐avuncular). Of 75 flies sampled from target mockingbird nests, 12 had at least one relative in a source mockingbird nest (16%).

We calculated 1065 kinship coefficients for pairs of flies from target finch nests and source mockingbird nests. Each fly from a target finch nest was compared to an average of 25.36 flies (range: 7–41) from source mockingbird nests to calculate kinship coefficients. Seven coefficients indicated second‐degree relatives (presumably avuncular), and 38 coefficients indicated third‐degree relatives (presumably half‐avuncular). Of 42 flies sampled from target finch nests, 15 had at least one relative in a source mockingbird nest (35.7%).

**FIGURE 5:**
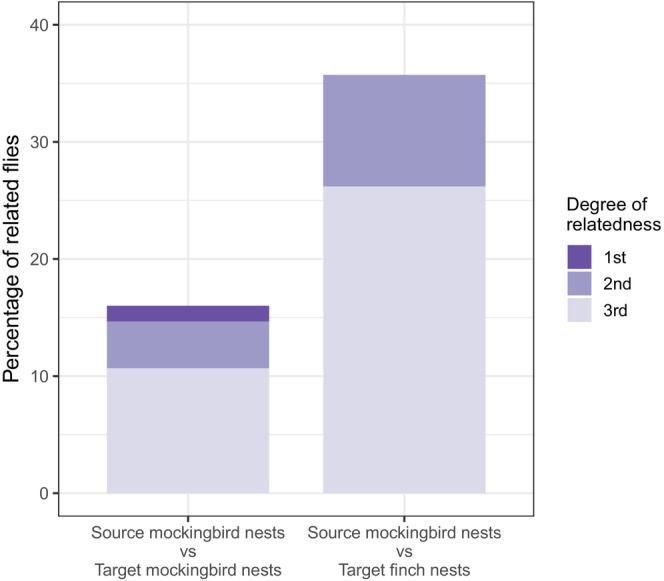
Stacked bar graph showing the percentage of flies from target mockingbird nests and target finch nests that are related to flies from source mockingbird nests. Shading shows the degree of relatedness. The percentage of related flies that were related to source mockingbird nests was significantly higher for target finch nests than for target mockingbird nests (*p* = 0.0279).

The percent of flies from target finch nests related to flies from source mockingbird nests, 35.7% (15/42), was significantly higher than the percent of flies from target mockingbird nests that were related to flies from source mockingbird nests, 16% (12/75) (Figure 5, *χ* ^2^ = 4.83, df = 1, *p* = 0.028).

## 4 Discussion

We used kinship analysis to infer whether avian vampire flies move from mockingbird nests to finch nests. Our results indicate that mockingbird nests are a source of flies for finch nests. Thirty‐five percent of flies in finch nests were closely related to flies from source mockingbird nests. In contrast, only 16% of flies in target mockingbird nests were closely related to flies from source mockingbird nests. These results suggest that flies that emerge from mockingbird nests lay eggs in later nesting finch nests more often than later nesting mockingbird nests. To our knowledge, this is the first demonstration of the role Galapagos mockingbirds play as a source of flies for Darwin's finches. Below we discuss two traits of mockingbirds that may increase their role as a source of flies, hypotheses to explain our observed patterns of relatedness, other potential sources of flies, and conservation implications.

The difference in timing of the breeding season between mockingbirds and finches may increase the indirect threat that mockingbird nests pose as a source of flies for finch nests. Mockingbirds typically start breeding earlier than finches. In 2022, the first mockingbird nest hatched 46 days before the first finch nest. It takes a minimum of 29 days for flies to go from an egg to an adult female fly capable of laying another generation of eggs (Causton et al. 2019). The difference in the timing of the breeding season of mockingbirds and finches therefore suggests there is ample time for flies emerging from mockingbird nests to lay eggs in finch nests.

Vampire flies are known to parasitize other avian hosts, such as Galapagos flycatchers ( *Myiarchus magnirostris* ), Galapagos doves ( *Zenaida galapagoensis* ) and smooth‐billed anis ( *Crotophaga ani* ) (Fessl and Tebbich 2002; Lincango et al. 2015; Coloma et al. 2020). Despite careful searching throughout El Garrapatero, we only found one nest that was not a mockingbird or a finch nest, a Galapagos flycatcher nest that hatched in late April, too late in the season to be a source of flies for target finch or mockingbird nests in our study. Future work in areas with greater host diversity could explore the role of other species as sources of flies for finches.

In our study, mockingbird nests had about twice as many flies in their nests as finches (Figure 4). Other studies have also found that mockingbird nests often have more flies than finch nests (Koop et al. 2011; Knutie et al. 2016). However, fly density (i.e., the number of flies per gram of host tissue) does not differ significantly between mockingbird and finch nests (Knutie et al. 2016). Mockingbird nests may have more flies because mockingbird nestlings are larger than finch nestlings, thus providing fly larvae larger amounts of resources. The substantially higher number of flies in mockingbird nests may increase the indirect threat of mockingbird nests as sources of flies. Vampire fly abundance generally increases as the breeding season progresses (Common et al. 2022). Thus, even relatively early nesting finches may suffer high abundance of vampire flies due to the earlier nesting mockingbirds which often have large number of flies.

Within source mockingbird nests, a majority of the flies were closely related. Of the 41 flies sampled in source nests, 22 had at least one half‐sibling in the same nest. We did not detect any full siblings within source nests. This is not entirely surprising as female vampire flies are known to mate with multiple males (Dudaniec et al. 2010); thus, a single clutch of fly eggs is likely to be composed of many half‐siblings. Not all of the flies in a single source nest were related to each other, which supports the finding by Dudaniec et al. (2010) that fly eggs in a single bird nest are often laid by several females.

Notably, flies in target mockingbird nests and target finch nests were related to flies in source mockingbird nests, consistent with transmission from source nests to target nests. The proportion of related flies was significantly higher between source mockingbird nests and target finch nests than between source and target mockingbird nests (Figure 5). This suggests that flies emerging from source mockingbird nests may more frequently lay eggs in target finch nests than in target mockingbird nests. This pattern may be driven by the distance between nests. In our study, target finch nests were denser than target mockingbird nests, which meant that the closest target nest to a source mockingbird nest was usually a finch nest. On average, finch nests were fairly close to the nearest source mockingbird nest (mean distance = 180 m). In comparison, the distance between target mockingbird nests and the nearest source mockingbird nest was over 50% farther (mean distance = 275 m). Fly transmission may occur on a relatively small geographic scale. Kleindorfer and Dudaniec (2009) found that the number of flies in the nests of small tree finches ( *Camarhynchus parvulus* ) is positively correlated with the density of nearby finch nests (Kleindorfer and Dudaniec 2009). Similarly, our data suggest that flies emerging from source mockingbird nests are likely to encounter and lay eggs in nearby finch and mockingbird nests.

Although we found considerable numbers of flies that were related between source and target nests, many flies in target nests (either mockingbird or finch) were not closely related to flies sequenced from source mockingbird nests. It is likely that our analyses underestimate the true number of relatives between nests because we only sequenced a subset of flies from a subset of nests. It is also likely that some of the flies in target nests may have come from other sources of flies such as nests outside of the study area and nests from the previous breeding season.

Our results are not consistent with preferential infestation of tolerant hosts. Unlike *Philornis torquans*, which prefer to infest tolerant hosts (Manzoli et al. 2021), our data suggest that avian vampire flies (*P. downsi*) do not prefer to infest tolerant hosts. Two differences between *P. torquans* and *P. downsi* may explain this difference. First, avian vampire flies are invasive and have a relatively short evolutionary history with Galapagos hosts, compared to the evolutionary history of *P. torquans* with Argentine hosts. In contrast, there may not be enough shared evolutionary history between avian vampire flies and Galapagos hosts for preference to have evolved. Second, Galapagos avifauna are not resistant to *P. downsi*. The preference of *P. torquans* to infest tolerant hosts may occur due to the presence of resistant hosts and vulnerable hosts, as well as tolerant hosts. For example, little thornbirds ( *Phacellodomus sibilatrix* ) are resistant to *P. torquans* and kill 82% of the larvae that infest them (Manzoli et al. 2018). Resistant little thornbirds are closely related to the vulnerable species, greater thornbirds ( *P. ruber* ). Consequently, the preference *P. torquans* has for tolerant great kiskadees ( *Pitangus sulphuratus* ) may be driven by selection to avoid infesting species of thornbirds (either little or greater) because flies infesting little thornbirds have low fitness and *P. torquans* cannot distinguish the two thornbird species (Manzoli et al. 2021). The Galapagos avifauna do not appear to have any resistant host species exerting selection on vampire flies for host preference.

Managing *P. downsi* is important because it is a major threat to Darwin's finches and causes up to 100% finch nestling mortality in some years (Koop et al. 2013). Even common species of Darwin's finches could be driven locally extinct within the next century (Koop et al. 2016). While initially counterintuitive, fumigating tolerant mockingbird nests may help Darwin's finches by reducing the number of flies in nests that are sources of flies for finches. Our data suggest that fumigation of mockingbird nests prior to the start of finch breeding might reduce the number of flies subsequently infesting finch nests. Targeted fumigation of mockingbirds could be used in combination with other management strategies, such as self‐fumigation by finches with permethrin‐treated cotton (Knutie et al. 2014). These short‐term solutions could conceivably reduce the impact of flies on finch populations, pending the development of long‐term strategies, such as biocontrol with parasitoid wasps (Bulgarella et al. 2017). Identification and targeted fumigation of sources of *Philornis* flies might also be used to help other endangered bird species threatened by other species of *Philornis*, such as Ridgway's hawk ( *Buteo ridgwayi* , Hayes et al. 2019) in the Dominican Republic, and the Puerto Rican Sharp‐shinned hawk ( *Accipiter striatus venator* , Delannoy and Cruz 1991) and the endemic Puerto Rican Parrot ( *Amazona vittata* , Snyder et al. 1987).

In conclusion, our data suggest three factors which increase the likelihood that infested mockingbird nests amplify the threat of avian vampire flies to finches. First, mockingbirds often start nesting earlier than finches. Second, mockingbird nests have nearly twice as many flies as finch nests. Third, flies infesting mockingbird nests are related to flies infesting finch nests. These results strongly suggest that Galapagos mockingbirds are an important source of parasitic flies that infest Darwin's finches.

More broadly, our study shows that kinship analysis is useful for inferring fine scale transmission in systems where other population genetics approaches may not be effective. Genetic approaches have been used to describe the pattern of transmission of other parasites and pathogens, such as SARS‐CoV‐2 (Lythgoe et al. 2021), but are usually limited to pathogens that evolve rapidly. For example, many viruses evolve rapidly due to high mutation rates, fast generation times, and large population sizes. The frequency and direction of transmission among hosts can be tracked by identifying viral strains among hosts. Our results suggest that kinship analysis can be useful to identify the frequency and direction of transmission in systems that do not evolve as quickly.

## Author Contributions

M.M.W., D.H.C., S.E.B. and A.D.S. designed the study and wrote the manuscript. M.M.W., D.H.C. and S.E.B. collected the data. M.M.W. analysed the data.

## Funding

This work was supported by the National Science Foundation, DEB‐2025085.

## Disclosure

Benefit Sharing Statement: Benefits Generated: This research helps to describe the transmission of an invasive parasite, *P. downsi*, which threatens Darwin's finches. This research identifies Galapagos mockingbirds as a source of *P. downsi* that could be targeted for control of *P. downsi* before they infest finches. This research generated 136 whole genomes of *P. downsi* that can be used in future genomic studies of *P. downsi*.

## Conflicts of Interest

The authors declare no conflicts of interest.

## Supporting information


**Appendix S1:** Illustrates how detection of avuncular or half‐avuncular kin between two nests is consistent with transmission of flies from the source nest to the target nest.
**Appendix S2:** describes the modified DNA extraction protocol used in this study.

## Data Availability

Genetic data: Raw sequence data will be uploaded to the GenBank SRA database pending article acceptance. Sample metadata: Related metadata and files used for analyses will be available in the Dryad Digital Repository pending article acceptance.

## References

[mec70334-bib-0001] Adelman, J. S. , A. W. Carter , W. A. Hopkins , and D. M. Hawley . 2013. “Deposition of Pathogenic *Mycoplasma gallisepticum* Onto Bird Feeders: Host Pathology Is More Important Than Temperature‐Driven Increases in Food Intake.” Biology Letters 9: 20130594. 23966599 10.1098/rsbl.2013.0594 PMC3971706

[mec70334-bib-0002] Basnet, A. , C. Palacios , H. Meng , et al. 2025. “Genomic Insights Into the Successful Invasion of the Avian Vampire Fly (*Philornis downsi*) in the Galapagos Islands.” Molecular Biology and Evolution 42: 1–13. 10.1093/molbev/msaf052 PMC11951964 40151837

[mec70334-bib-0003] Bolger, A. M. , M. Lohse , and B. Usadel . 2014. “Trimmomatic: A Flexible Trimmer for Illumina Sequence Data.” Bioinformatics 30: 2114–2120. 24695404 10.1093/bioinformatics/btu170 PMC4103590

[mec70334-bib-0004] Bulgarella, M. , M. P. Lincango , P. F. Lahuatte , et al. 2022. “Persistence of the Invasive Bird‐Parasitic Fly *Philornis downsi* Over the Host Interbreeding Period in the Galapagos Islands.” Scientific Reports 12: 2325. 35149738 10.1038/s41598-022-06208-5 PMC8837626

[mec70334-bib-0005] Bulgarella, M. , M. A. Quiroga , R. A. Boulton , et al. 2017. “Life Cycle and Host Specificity of the Parasitoid *Conura annulifera* (Hymenoptera: Chalcididae), a Potential Biological Control Agent of *Philornis downsi* (Diptera: Muscidae) in the Galapagos Islands.” Annals of the Entomological Society of America 110: 317–328.

[mec70334-bib-0006] Causton, C. E. , R. D. Moon , A. Cimadom , et al. 2019. “Population Dynamics of an Invasive Bird Parasite, *Philornis downsi* (Diptera: Muscidae), in the Galapagos Islands.” PLoS One 14: e0224125. 31626686 10.1371/journal.pone.0224125 PMC6874344

[mec70334-bib-0007] Causton, C. E. , S. B. Peck , B. J. Sinclair , L. Roque‐Albelo , C. J. Hodgson , and B. Landry . 2006. “Alien Insects: Threats and Implications for Conservation of Galapagos Islands.” Annals of the Entomological Society of America 99: 121–143.

[mec70334-bib-0008] Coloma, A. , D. Anchundia , P. Piedrahita , C. Pike , and B. Fessl . 2020. “Observations on the Nesting of the Galapagos Dove *Zenaida galapagoensis* in Galapagos, Ecuador.” Galapagos Research 69: 34–38.

[mec70334-bib-0009] Common, L. K. , P. Sumasgutner , S. C. Sumasgutner , D. Colombelli‐Négrel , R. Y. Dudaniec , and S. Kleindorfer . 2022. “Temporal and Spatial Variation in Sex‐Specific Abundance of the Avian Vampire Fly (*Philornis downsi*).” Parasitology Research 121: 63–74. 34799771 10.1007/s00436-021-07350-1 PMC8748338

[mec70334-bib-0010] Danecek, P. , A. Auton , G. Abecasis , et al. 2011. “The Variant Call Format and VCFtools.” Bioinformatics 27: 2156–2158. 21653522 10.1093/bioinformatics/btr330 PMC3137218

[mec70334-bib-0011] Danecek, P. , J. K. Bonfield , J. Liddle , et al. 2021. “Twelve Years of SAMtools and BCFtools.” GigaScience 10: 1–4. 10.1093/gigascience/giab008 PMC7931819 33590861

[mec70334-bib-0012] Delannoy, C. A. , and A. Cruz . 1991. “ *Philornis* Parasitism and Nestling Survival of the Puerto Rican Sharp‐Shinned Hawk.” In Bird–Parasite Interactions. Ecology, Evolution and Behaviour, edited by J. Loye and M. Zuk . Oxford University Press.

[mec70334-bib-0013] Dudaniec, R. Y. , M. G. Gardner , and S. Kleindorfer . 2010. “Offspring Genetic Structure Reveals Mating and Nest Infestation Behaviour of an Invasive Parasitic Fly (*Philornis downsi*) of Galápagos Birds.” Biological Invasions 12: 581–592.

[mec70334-bib-0014] Fessl, B. , G. E. Heimpel , and C. E. Causton . 2018. “Invasion of an Avian Nest Parasite, *Philornis downsi*, to the Galapagos Islands: Colonization History, Adaptations to Novel Ecosystems, and Conservation Challenges.” In Disease Ecology, Social and Ecological Interactions in the Galapagos Islands, edited by P. G. Parker . Springer International Publishing.

[mec70334-bib-0015] Fessl, B. , B. J. Sinclair , and S. Kleindorfer . 2006. “The Life‐Cycle of *Philornis downsi* (Diptera: Muscidae) Parasitizing Darwin's Finches and Its Impacts on Nestling Survival.” Parasitology 133: 739–747. 16899139 10.1017/S0031182006001089

[mec70334-bib-0016] Fessl, B. , and S. Tebbich . 2002. “ *Philornis downsi* – A Recently Discovered Parasite on the Galapagos Archipelago – A Threat for Darwin's Finches?” Ibis 144: 445–451.

[mec70334-bib-0017] Fessl, B. , G. H. Young , R. P. Young , et al. 2010. “How to Save the Rarest Darwin's Finch From Extinction: The Mangrove Finch on Isabela Island.” Philosophical Transactions of the Royal Society of London B 365: 1019–1030. 10.1098/rstb.2009.0288 PMC2830234 20194165

[mec70334-bib-0018] Grant, P. R. 1999. Ecology and Evolution of Darwin's Finches. Princeton University Press.

[mec70334-bib-0019] Hanghøj, K. , I. Moltke , P. A. Anderson , A. Manica , and T. S. Korneliussen . 2019. “Fast and Accurate Relatedness Estimates From High Throughput Sequencing Data in the Presence of Inbreeding.” GigaScience 8: 1–9. 10.1093/gigascience/giz034 PMC6488770 31042285

[mec70334-bib-0020] Hayes, C. D. , T. I. Hayes , C. J. W. McClure , M. Quiroga , R. K. Thorstrom , and D. L. Anderson . 2019. “Native Parasitic Nest Fly Impacts Reproductive Success of an Island‐Endemic Host.” Animal Conservation 22: 157–164.

[mec70334-bib-0021] Henschen, A. E. , and J. S. Adelman . 2019. “What Does Tolerance Mean for Animal Disease Dynamics When Pathology Enhances Transmission?” Integrative and Comparative Biology 59: 1220–1230. 31141137 10.1093/icb/icz065

[mec70334-bib-0022] Jacquard, A. 1974. The Genetic Structure of Populations. Springer.

[mec70334-bib-0023] Jasper, M. , T. L. Schmidt , N. W. Ahmad , S. P. Sinkins , and A. A. Hoffmann . 2019. “A Genomic Approach to Inferring Kinship Reveals Limited Intergenerational Dispersal in the Yellow Fever Mosquito.” Molecular Ecology Resources 92: 1254–1265. 10.1111/1755-0998.13043 PMC6790672 31125998

[mec70334-bib-0024] Kleindorfer, S. , and R. Y. Dudaniec . 2009. “Love Thy Neighbor? Social Nesting Pattern, Host Mass and Nest Size Affect Ectoparasite Intensity in Darwin's Tree Finches.” Behavioral Ecology and Sociobiology 63: 731–739.

[mec70334-bib-0025] Knutie, S. A. , S. M. McNew , A. W. Bartlow , D. A. Vargas , and D. H. Clayton . 2014. “Darwin's Finches Combat Introduced Nest Parasites With Fumigated Cotton.” Current Biology 24: R355–R356. 24801182 10.1016/j.cub.2014.03.058

[mec70334-bib-0026] Knutie, S. A. , J. P. Owen , S. M. McNew , et al. 2016. “Galápagos Mockingbirds Are Tolerant Hosts of Introduced Parasites That Threaten Darwin's Finches.” Ecology 97: 940–950. 27220210

[mec70334-bib-0027] Knutie, S. A. , C. N. Webster , G. J. Vaziri , et al. 2024. “Urban Living Can Rescue Darwin's Finches From the Lethal Effects of Invasive Vampire Flies.” Global Change Biology 30: e17145. 38273516 10.1111/gcb.17145

[mec70334-bib-0028] Koop, J. A. H. , S. K. Huber , S. M. Laverty , and D. H. Clayton . 2011. “Experimental Demonstration of the Fitness Consequences of an Introduced Parasite of Darwin's Finches.” PLoS One 6: e19706. 21589659 10.1371/journal.pone.0019706 PMC3092749

[mec70334-bib-0029] Koop, J. A. H. , P. S. Kim , S. A. Knutie , F. Adler , and D. H. Clayton . 2016. “Introduced Parasitic Fly May Lead to Local Extinction of Darwin's Finch Populations.” Journal of Applied Ecology 53: 511–518. 26980922 10.1111/1365-2664.12575 PMC4788638

[mec70334-bib-0030] Koop, J. A. H. , J. P. Owen , S. A. Knutie , M. A. Aguilar , and D. H. Clayton . 2013. “Experimental Demonstration of a Parasite‐Induced Immune Response in Wild Birds: Darwin's Finches and Introduced Nest Flies.” Ecology and Evolution 3: 2514–2523. 24567824 10.1002/ece3.651 PMC3930052

[mec70334-bib-0031] Langmead, B. , and S. Salzberg . 2012. “Fast Gapped‐Read Alignment With Bowtie 2.” Nature Methods 9: 357–359. 22388286 10.1038/nmeth.1923 PMC3322381

[mec70334-bib-0032] Li, H. 2011. “A Statistical Framework for SNP Calling, Mutation Discovery, Association Mapping and Population Genetical Parameter Estimation From Sequencing Data.” Bioinformatics 27, no. 2987: 2993. 10.1093/bioinformatics/btr509 PMC3198575 21903627

[mec70334-bib-0033] Lincango, P. , C. Causton , D. Cedeño , J. Castañeda , A. Hillstrom , and D. Freund . 2015. “Interactions Between the Avian Parasite, *Philornis downsi* (Diptera: Muscidae) and the Galapagos Flycatcher, *Myiarchus magnirostris* Gould (Passeriformes: Tyrannidae).” Journal of Wildlife Diseases 51: 907–910. 26267462 10.7589/2015-01-025

[mec70334-bib-0034] Lomas, E. 2008. “Dispersión de insectos por las luces de los barcos en las islas Galápagos: Una prioridad de conservación.” Undergraduate thesis. Universidad Central del Ecuador y Fundación Charles Darwin.

[mec70334-bib-0035] Lythgoe, K. A. , M. Hall , L. Ferretti , et al. 2021. “SARS‐CoV‐2 Within‐Host Diversity and Transmission.” Science 372: eabg0821. 33688063 10.1126/science.abg0821 PMC8128293

[mec70334-bib-0036] Manzoli, D. E. , L. R. Antoniazzi , M. J. Saravia , L. Silvestri , D. Rorhmann , and P. M. Beldomenico . 2013. “Multi‐Level Determinants of Parasitic Fly Infection in Forest Passerines.” PLoS One 8: e67104. 23874408 10.1371/journal.pone.0067104 PMC3707910

[mec70334-bib-0037] Manzoli, D. E. , M. J. Saravia‐Pietropaolo , L. R. Antoniazzi , et al. 2018. “Contrasting Consequences of Different Defence Strategies in a Natural Multihost‐Parasite System.” International Journal for Parasitology 48: 445–455. 29391194 10.1016/j.ijpara.2017.11.001

[mec70334-bib-0038] Manzoli, D. E. , M. J. Saravia‐Pietropaolo , S. I. Arce , A. Percara , L. R. Antoniazzi , and P. M. Beldomenico . 2021. “Specialist by Preference, Generalist by Need: Availability of Quality Hosts Drives Parasite Choice in a Natural Multihost–Parasite System.” International Journal for Parasitology 51: 527–534. 33713648 10.1016/j.ijpara.2020.12.003

[mec70334-bib-0039] Martin, L. B. , B. Addison , A. G. D. Bean , et al. 2019. “Extreme Competence: Keystone Hosts of Infections.” Trends in Ecology and Evolution 34: 303–314. 30704782 10.1016/j.tree.2018.12.009 PMC7114649

[mec70334-bib-0040] McNew, S. M. , S. A. Knutie , G. B. Goodman , et al. 2019. “Annual Environmental Variation Influences Host Tolerance to Parasites.” Proceedings of the Royal Society B 286: 20190049. 30963843 10.1098/rspb.2019.0049 PMC6408884

[mec70334-bib-0041] Medzhitov, R. , D. S. Schneider , and M. P. Soares . 2012. “Disease Tolerance as a Defense Strategy.” Science 335: 936–941. 22363001 10.1126/science.1214935 PMC3564547

[mec70334-bib-0042] Miller, M. R. , A. White , and M. Boots . 2005. “The Evolution of Host Resistance: Tolerance and Control as Distinct Strategies.” Journal of Theoretical Biology 236: 198–207. 16005309 10.1016/j.jtbi.2005.03.005

[mec70334-bib-0043] O'Connor, J. A. , F. J. Sulloway , J. Robertson , and S. Kleindorfer . 2009. “ *Philornis downsi* Parasitism Is the Primary Cause of Nestling Mortality in the Critically Endangered Darwin's Medium Tree Finch (*Camarhynnchus pauper*).” Biodiversity and Conservation 19: 853–866.

[mec70334-bib-0044] R Core Team . 2024. R: A Language for Statistical Computing. R Foundation for Statistical Computing.

[mec70334-bib-0045] Romine, M. G. , S. A. Knutie , C. M. Crow , et al. 2022. “The Genome Sequence of the Avian Vampire Fly (*Philornis downsi*), an Invasive Nest Parasite of Darwin's Finches in Galapagos.” G3: Genes, Genomes, Genetics 12: 1–9. 10.1093/g3journal/jkab414 PMC9210292 34878103

[mec70334-bib-0046] Roy, B. A. , and J. W. Kirchner . 2000. “Evolutionary Dynamics of Pathogen Resistance and Tolerance.” Evolution 54: 51–63. 10937183 10.1111/j.0014-3820.2000.tb00007.x

[mec70334-bib-0047] Scheele, B. C. , D. A. Hunter , L. A. Brannelly , L. F. Skerratt , and D. A. Driscoll . 2017. “Reservoir‐Host Amplification of Disease Impact in an Endangered Amphibian.” Conservation Biology 31: 592–600. 27594575 10.1111/cobi.12830

[mec70334-bib-0048] Schmidt, T. L. , S. Elfekih , L. Cao , et al. 2023. “Close Kin Dyads Indicate Intergenerational Dispersal and Barriers.” American Naturalist 201: 65–77. 10.1086/722175 36524932

[mec70334-bib-0049] Snyder, N. F. R. , J. W. Wiley , and C. B. Kepler . 1987. The Parrots of Luquillo: Natural History and Conservation of the Puerto Rican Parrot. Western Foundation of Vertebrate Zoology.

[mec70334-bib-0050] Tadiri, C. P. , G. F. Fussman , and M. E. Scott . 2021. “Parasite Spread in Experimental Metapopulations: Resistance, Tolerance and Host Competence.” Oikos 130: 1562–1571.

[mec70334-bib-0051] Tompkins, D. M. , A. R. White , and M. Boots . 2003. “Ecological Replacement of Native Red Squirrels by Invasive Greys Driven by Disease.” Ecology Letters 6: 189–196.

[mec70334-bib-0052] VanderWaal, K. L. , and V. O. Ezenwa . 2016. “Heterogeneity in Pathogen Transmission.” Functional Ecology 30: 1606–1622.

[mec70334-bib-0053] Wiedenfeld, D. A. , B. Fessl , S. Kleindorfer , and J. C. Valarezo . 2007. “Distribution of the Introduced Parasitic Fly *Philornis downsi* (Diptera, Muscidae) in the Galapagos Islands.” Pacific Conservation Biology 13: 14–19.

