# Supplementary material for: Kinship Analysis Confirms Tolerant Galapagos Mockingbirds Are a Source of Nest Flies That Threaten Darwin's Finches
Source: Mol Ecol. 2026 Apr 2;35(7):e70334. doi: 10.1111/mec.70334 (PMC13044697; doi:10.1111/mec.70334)
Supplement: Supplementary file 1 — Appendix S1: Illustrates how detection of avuncular or half‐avuncular kin between two nests is consistent with transmission of flies from the source nest to the target nest. Appendix S2: describes the modified DNA extraction protocol used in this study. [file MEC-35-e70334-s001.docx]

**Supplemental material:**

Supplement 1. Inferring fly transmission using kinship data.

Supplemental Figure 1. Conceptual figure showing how sampling of avuncular kin from two nests is consistent with transmission of flies from the source nest to the target nest.


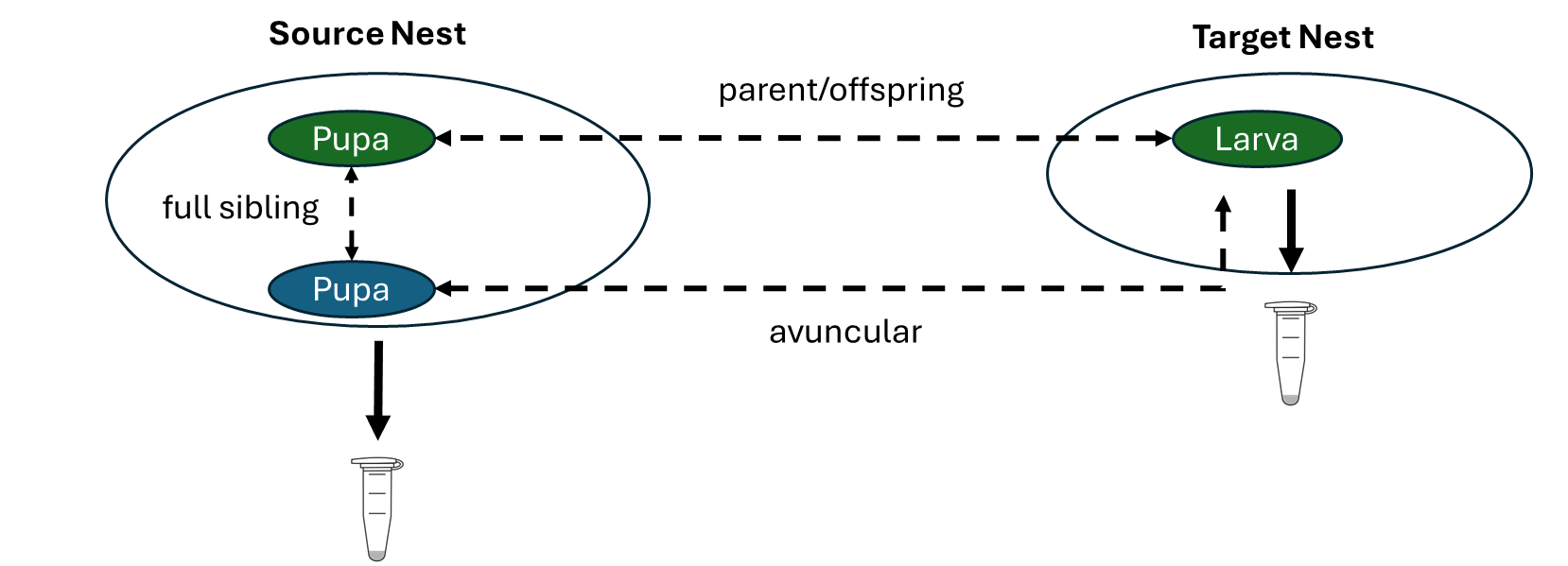


Colored ovals represent individual flies. Dashed lines show degrees of relatedness. Consider a source nest with a pair of full siblings, a green pupa and blue pupa. Assume the blue pupa is preserved for sequencing, while the green pupa is allowed to eclose into an adult fly, which lays eggs in the target nest. The blue pupa is the uncle or aunt of the larva, representing an avuncular relationship. We infer the direction of transmission by searching for relatives that are separated by one generation. Flies in target nests were likely to be one generation after flies in source nests because flies in target nests were preserved at least 29 days (the minimum fly generation time) after flies in source nests were preserved. Thus, an avuncular relationship between a fly in a source nest and a fly in a target nest is evidence of transmission from the source nest to the target nest.

Supplemental Figure 2. Conceptual figure showing how sampling of half-avuncular kin from two nests is consistent with transmission of flies from the source nest to the target nest.


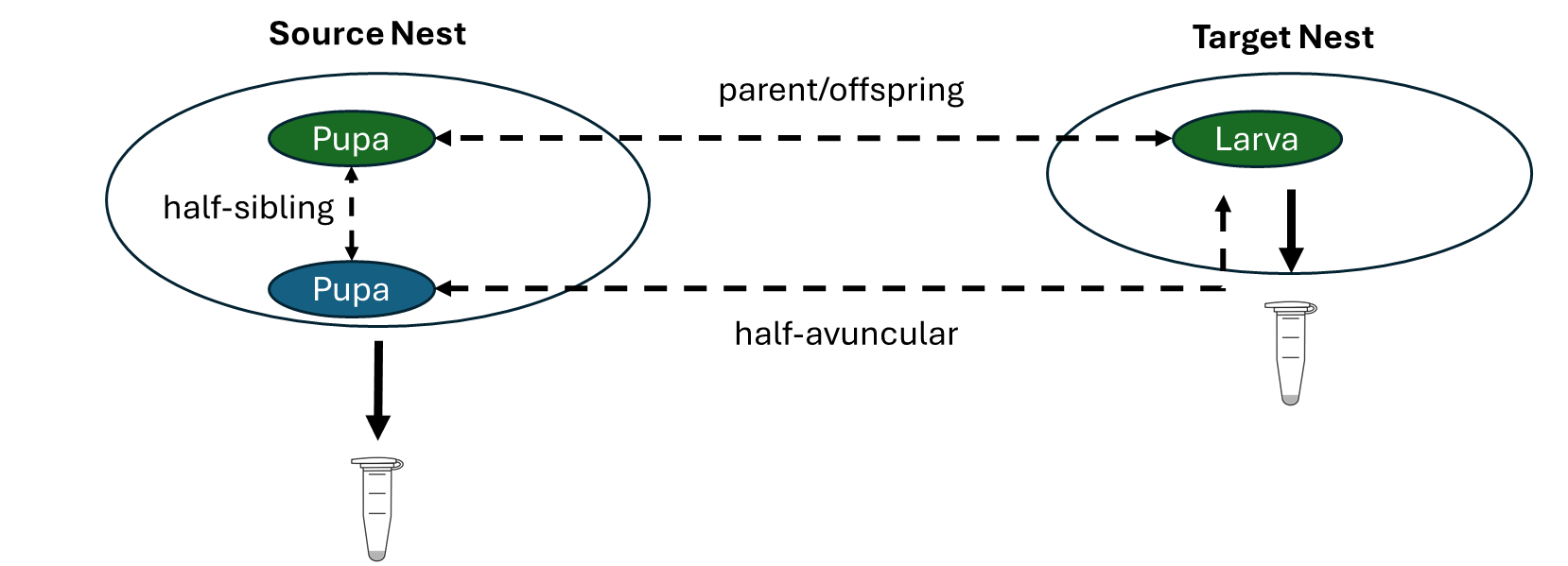


Colored ovals represent individual flies. Dashed lines show degrees of relatedness. Consider a source nest with a pair of half-siblings, a green pupa and blue pupa. Assume the blue pupa is preserved for sequencing, while the green pupa is allowed to eclose into an adult fly, which lays eggs in the target nest. The blue pupa is the half-uncle or half-aunt of the larva, representing a half-avuncular relationship (offspring of a half-sibling). We infer the direction of transmission by searching for relatives that are separated by one generation. Flies in target nests were likely to be one generation after flies in source nests because flies in target nests were preserved at least 29 days (the minimum fly generation time) after flies in source nests were preserved. Thus, a half-avuncular relationship between a fly in a source nest and a fly in a target nest is evidence of transmission from the source nest to the target nest.

Supplement 2. *Philornis downsi* extraction protocol

**DNA Extraction Protocol**

Adapted from Qiagen DNeasy kit for *Philornis downs*i larvae and pupae

**Materials and Instruments:**

- Qiagen DNeasy Blood and Tissue Kit (category no. 69504)
- Tabletop incubator with shaking supplies
- Centrifuge
- Vortexer
- Tweezers
- Dissection scissors
- 1,000 uL pipettor and tips
- 100 uL pipettor and tips
- Microcentrifuge tubes
- 96-100% ethanol

**Protocol:**

Disinfect before starting (10% bleach, 70% ethanol, DNA away)

**Day 1**

1. Preheat incubator to 56° C
2. Cut larval or pupal sample in half (will only be using half for extraction, other half will be preserved as a voucher)
3. Cut up one half of the sample into small pieces and place into microcentrifuge tube. Disinfect scissors and tweezers and change gloves between samples.
4. Add 40 uL Proteinase K
5. Vortex
6. 180 uL Buffer ATL
7. Vortex
8. Put in incubator with shaking function on at 56° C for 24 hours

**Day 2**

1. Take samples out of incubator, and set incubator to 37° C
2. Vortex
3. Centrifuge for 3 minutes at 14,000 rpm
4. Pipette supernatant into a new microcentrifuge tube
5. Add 220 uL Buffer AL
6. Vortex
7. Add 220 uL Ethanol
8. Vortex
9. Pipette into spin column with tube
10. Centrifuge for 1 minute at 8,000 rpm
11. Discard flow through
12. Add 500 uL Buffer AW1 (Note: make sure ethanol has been added to buffer)
13. Centrifuge for 1 minute at 8,000 rpm
14. Discard flow through
15. Add 500 uL Buffer AW2 (Note: make sure ethanol has been added to buffer)
16. Centrifuge for 3 minute at 14,000 rpm
17. Place spin column into a new microcentrifuge tube
18. Add 100 uL Buffer AE onto membrane
19. Incubate at 37° C for 5 minutes
20. Centrifuge for 1 minute at 8,000 rpm
21. Flow through is first elution, label microcentrifuge tube
22. Repeat steps 17-20 for second elution, label microcentrifuge tube
